# Supplementary material for: HIVprotI: an integrated web based platform for prediction and design of HIV proteins inhibitors
Source: J Cheminform. 2018 Mar 9;10:12. doi: 10.1186/s13321-018-0266-y (PMC5845081; doi:10.1186/s13321-018-0266-y)
Supplement: Supplementary file 2 — Additional file 2. Source code of HIVProtI web server. [file 13321_2018_266_MOESM2_ESM.zip › HIVprotI_Source-code/hivprotI/weka-3-6-6/doc/weka/gui/arffviewer/ArffTable.html]

ArffTable


---


|  |  |  |
| --- | --- | --- |
| **Overview** \| **Package** \| **Class** \| **Tree** \| **Deprecated** \| **Index** \| **Help** \| **Weka's home** | |  |
| **PREV CLASS**   **NEXT CLASS** | **FRAMES**    **NO FRAMES**     **All Classes** |  |
| SUMMARY: NESTED \| FIELD \| CONSTR \| METHOD | DETAIL: FIELD \| CONSTR \| METHOD |  |


---


## weka.gui.arffviewer Class ArffTable

```
java.lang.Object
  java.awt.Component
      java.awt.Container
          javax.swing.JComponent
              javax.swing.JTable
                  weka.gui.arffviewer.ArffTable
```

**All Implemented Interfaces:**: java.awt.image.ImageObserver, java.awt.MenuContainer, java.io.Serializable, java.util.EventListener, javax.accessibility.Accessible, javax.swing.event.CellEditorListener, javax.swing.event.ListSelectionListener, javax.swing.event.RowSorterListener, javax.swing.event.TableColumnModelListener, javax.swing.event.TableModelListener, javax.swing.Scrollable

---

``` public class ArffTable extends javax.swing.JTable ```

A specialized JTable for the Arff-Viewer.

**Version:**
:   $Revision: 7059 $

**Author:**
:   FracPete (fracpete at waikato dot ac dot nz)

**See Also:**: Serialized Form

---

**Nested Class Summary**

**Nested classes/interfaces inherited from class javax.swing.JTable**

`javax.swing.JTable.DropLocation, javax.swing.JTable.PrintMode`

**Nested classes/interfaces inherited from class javax.swing.JComponent**

`javax.swing.JComponent.AccessibleJComponent`

**Nested classes/interfaces inherited from class java.awt.Component**

`java.awt.Component.BaselineResizeBehavior`


**Field Summary**

**Fields inherited from class javax.swing.JTable**

`AUTO_RESIZE_ALL_COLUMNS, AUTO_RESIZE_LAST_COLUMN, AUTO_RESIZE_NEXT_COLUMN, AUTO_RESIZE_OFF, AUTO_RESIZE_SUBSEQUENT_COLUMNS`

**Fields inherited from class javax.swing.JComponent**

`TOOL_TIP_TEXT_KEY, UNDEFINED_CONDITION, WHEN_ANCESTOR_OF_FOCUSED_COMPONENT, WHEN_FOCUSED, WHEN_IN_FOCUSED_WINDOW`

**Fields inherited from class java.awt.Component**

`BOTTOM_ALIGNMENT, CENTER_ALIGNMENT, LEFT_ALIGNMENT, RIGHT_ALIGNMENT, TOP_ALIGNMENT`

**Fields inherited from interface java.awt.image.ImageObserver**

`ABORT, ALLBITS, ERROR, FRAMEBITS, HEIGHT, PROPERTIES, SOMEBITS, WIDTH`


**Constructor Summary**

`ArffTable()`
  
          initializes with no model

`ArffTable(javax.swing.table.TableModel model)`
  
          initializes with the given model


| **Method Summary** | |
| --- | --- |
| `void` | `addChangeListener(javax.swing.event.ChangeListener l)`             Adds a ChangeListener to the panel |
| `javax.swing.table.TableCellEditor` | `getCellEditor(int row, int column)`             returns the cell editor for the given cell |
| `java.lang.String` | `getPlainColumnName(int columnIndex)`             returns the basically the attribute name of the column and not the HTML column name via getColumnName(int) |
| `java.lang.String` | `getSearchString()`             returns the search string, can be NULL if no search string is set |
| `java.awt.datatransfer.StringSelection` | `getStringSelection()`             returns the selected content in a StringSelection that can be copied to the clipboard and used in Excel, if nothing is selected the whole table is copied to the clipboard |
| `boolean` | `isReadOnly()`             returns whether the model is read-only |
| `void` | `removeChangeListener(javax.swing.event.ChangeListener l)`             Removes a ChangeListener from the panel |
| `void` | `setModel(javax.swing.table.TableModel model)`             sets the new model |
| `void` | `setReadOnly(boolean value)`             sets whether the model is read-only |
| `void` | `setSearchString(java.lang.String searchString)`             sets the search string to look for in the table, NULL or "" disables the search |
| `void` | `setSelectedColumn(int index)`             sets the selected column |
| `void` | `tableChanged(javax.swing.event.TableModelEvent e)`             This fine grain notification tells listeners the exact range of cells, rows, or columns that changed. |

**Methods inherited from class javax.swing.JTable**

`addColumn, addColumnSelectionInterval, addNotify, addRowSelectionInterval, changeSelection, clearSelection, columnAdded, columnAtPoint, columnMarginChanged, columnMoved, columnRemoved, columnSelectionChanged, convertColumnIndexToModel, convertColumnIndexToView, convertRowIndexToModel, convertRowIndexToView, createDefaultColumnsFromModel, createScrollPaneForTable, doLayout, editCellAt, editCellAt, editingCanceled, editingStopped, getAccessibleContext, getAutoCreateColumnsFromModel, getAutoCreateRowSorter, getAutoResizeMode, getCellEditor, getCellRect, getCellRenderer, getCellSelectionEnabled, getColumn, getColumnClass, getColumnCount, getColumnModel, getColumnName, getColumnSelectionAllowed, getDefaultEditor, getDefaultRenderer, getDragEnabled, getDropLocation, getDropMode, getEditingColumn, getEditingRow, getEditorComponent, getFillsViewportHeight, getGridColor, getIntercellSpacing, getModel, getPreferredScrollableViewportSize, getPrintable, getRowCount, getRowHeight, getRowHeight, getRowMargin, getRowSelectionAllowed, getRowSorter, getScrollableBlockIncrement, getScrollableTracksViewportHeight, getScrollableTracksViewportWidth, getScrollableUnitIncrement, getSelectedColumn, getSelectedColumnCount, getSelectedColumns, getSelectedRow, getSelectedRowCount, getSelectedRows, getSelectionBackground, getSelectionForeground, getSelectionModel, getShowHorizontalLines, getShowVerticalLines, getSurrendersFocusOnKeystroke, getTableHeader, getToolTipText, getUI, getUIClassID, getUpdateSelectionOnSort, getValueAt, isCellEditable, isCellSelected, isColumnSelected, isEditing, isRowSelected, moveColumn, prepareEditor, prepareRenderer, print, print, print, print, print, removeColumn, removeColumnSelectionInterval, removeEditor, removeNotify, removeRowSelectionInterval, rowAtPoint, selectAll, setAutoCreateColumnsFromModel, setAutoCreateRowSorter, setAutoResizeMode, setCellEditor, setCellSelectionEnabled, setColumnModel, setColumnSelectionAllowed, setColumnSelectionInterval, setDefaultEditor, setDefaultRenderer, setDragEnabled, setDropMode, setEditingColumn, setEditingRow, setFillsViewportHeight, setGridColor, setIntercellSpacing, setPreferredScrollableViewportSize, setRowHeight, setRowHeight, setRowMargin, setRowSelectionAllowed, setRowSelectionInterval, setRowSorter, setSelectionBackground, setSelectionForeground, setSelectionMode, setSelectionModel, setShowGrid, setShowHorizontalLines, setShowVerticalLines, setSurrendersFocusOnKeystroke, setTableHeader, setUI, setUpdateSelectionOnSort, setValueAt, sizeColumnsToFit, sizeColumnsToFit, sorterChanged, updateUI, valueChanged`

**Methods inherited from class javax.swing.JComponent**

`addAncestorListener, addVetoableChangeListener, computeVisibleRect, contains, createToolTip, disable, enable, firePropertyChange, firePropertyChange, firePropertyChange, getActionForKeyStroke, getActionMap, getAlignmentX, getAlignmentY, getAncestorListeners, getAutoscrolls, getBaseline, getBaselineResizeBehavior, getBorder, getBounds, getClientProperty, getComponentPopupMenu, getConditionForKeyStroke, getDebugGraphicsOptions, getDefaultLocale, getFontMetrics, getGraphics, getHeight, getInheritsPopupMenu, getInputMap, getInputMap, getInputVerifier, getInsets, getInsets, getListeners, getLocation, getMaximumSize, getMinimumSize, getNextFocusableComponent, getPopupLocation, getPreferredSize, getRegisteredKeyStrokes, getRootPane, getSize, getToolTipLocation, getToolTipText, getTopLevelAncestor, getTransferHandler, getVerifyInputWhenFocusTarget, getVetoableChangeListeners, getVisibleRect, getWidth, getX, getY, grabFocus, isDoubleBuffered, isLightweightComponent, isManagingFocus, isOpaque, isOptimizedDrawingEnabled, isPaintingForPrint, isPaintingTile, isRequestFocusEnabled, isValidateRoot, paint, paintImmediately, paintImmediately, print, printAll, putClientProperty, registerKeyboardAction, registerKeyboardAction, removeAncestorListener, removeVetoableChangeListener, repaint, repaint, requestDefaultFocus, requestFocus, requestFocus, requestFocusInWindow, resetKeyboardActions, reshape, revalidate, scrollRectToVisible, setActionMap, setAlignmentX, setAlignmentY, setAutoscrolls, setBackground, setBorder, setComponentPopupMenu, setDebugGraphicsOptions, setDefaultLocale, setDoubleBuffered, setEnabled, setFocusTraversalKeys, setFont, setForeground, setInheritsPopupMenu, setInputMap, setInputVerifier, setMaximumSize, setMinimumSize, setNextFocusableComponent, setOpaque, setPreferredSize, setRequestFocusEnabled, setToolTipText, setTransferHandler, setVerifyInputWhenFocusTarget, setVisible, unregisterKeyboardAction, update`

**Methods inherited from class java.awt.Container**

`add, add, add, add, add, addContainerListener, addPropertyChangeListener, addPropertyChangeListener, applyComponentOrientation, areFocusTraversalKeysSet, countComponents, deliverEvent, findComponentAt, findComponentAt, getComponent, getComponentAt, getComponentAt, getComponentCount, getComponents, getComponentZOrder, getContainerListeners, getFocusTraversalKeys, getFocusTraversalPolicy, getLayout, getMousePosition, insets, invalidate, isAncestorOf, isFocusCycleRoot, isFocusCycleRoot, isFocusTraversalPolicyProvider, isFocusTraversalPolicySet, layout, list, list, locate, minimumSize, paintComponents, preferredSize, printComponents, remove, remove, removeAll, removeContainerListener, setComponentZOrder, setFocusCycleRoot, setFocusTraversalPolicy, setFocusTraversalPolicyProvider, setLayout, transferFocusBackward, transferFocusDownCycle, validate`

**Methods inherited from class java.awt.Component**

`action, add, addComponentListener, addFocusListener, addHierarchyBoundsListener, addHierarchyListener, addInputMethodListener, addKeyListener, addMouseListener, addMouseMotionListener, addMouseWheelListener, bounds, checkImage, checkImage, contains, createImage, createImage, createVolatileImage, createVolatileImage, dispatchEvent, enable, enableInputMethods, firePropertyChange, firePropertyChange, firePropertyChange, firePropertyChange, firePropertyChange, getBackground, getBounds, getColorModel, getComponentListeners, getComponentOrientation, getCursor, getDropTarget, getFocusCycleRootAncestor, getFocusListeners, getFocusTraversalKeysEnabled, getFont, getForeground, getGraphicsConfiguration, getHierarchyBoundsListeners, getHierarchyListeners, getIgnoreRepaint, getInputContext, getInputMethodListeners, getInputMethodRequests, getKeyListeners, getLocale, getLocation, getLocationOnScreen, getMouseListeners, getMouseMotionListeners, getMousePosition, getMouseWheelListeners, getName, getParent, getPeer, getPropertyChangeListeners, getPropertyChangeListeners, getSize, getToolkit, getTreeLock, gotFocus, handleEvent, hasFocus, hide, imageUpdate, inside, isBackgroundSet, isCursorSet, isDisplayable, isEnabled, isFocusable, isFocusOwner, isFocusTraversable, isFontSet, isForegroundSet, isLightweight, isMaximumSizeSet, isMinimumSizeSet, isPreferredSizeSet, isShowing, isValid, isVisible, keyDown, keyUp, list, list, list, location, lostFocus, mouseDown, mouseDrag, mouseEnter, mouseExit, mouseMove, mouseUp, move, nextFocus, paintAll, postEvent, prepareImage, prepareImage, remove, removeComponentListener, removeFocusListener, removeHierarchyBoundsListener, removeHierarchyListener, removeInputMethodListener, removeKeyListener, removeMouseListener, removeMouseMotionListener, removeMouseWheelListener, removePropertyChangeListener, removePropertyChangeListener, repaint, repaint, repaint, resize, resize, setBounds, setBounds, setComponentOrientation, setCursor, setDropTarget, setFocusable, setFocusTraversalKeysEnabled, setIgnoreRepaint, setLocale, setLocation, setLocation, setName, setSize, setSize, show, show, size, toString, transferFocus, transferFocusUpCycle`

**Methods inherited from class java.lang.Object**

`equals, getClass, hashCode, notify, notifyAll, wait, wait, wait`

**Constructor Detail**

### ArffTable

```
public ArffTable()
```

:   initializes with no model

---


### ArffTable

```
public ArffTable(javax.swing.table.TableModel model)
```

:   initializes with the given model

    **Parameters:**: `model` - the model to use


**Method Detail**

### setModel

```
public void setModel(javax.swing.table.TableModel model)
```

:   sets the new model

    :   **Overrides:**: `setModel` in class `javax.swing.JTable`
    :   **Parameters:**: `model` - the model to use

---


### getCellEditor

```
public javax.swing.table.TableCellEditor getCellEditor(int row,
                                                       int column)
```

:   returns the cell editor for the given cell

    :   **Overrides:**: `getCellEditor` in class `javax.swing.JTable`
    :   **Parameters:**: `row` - the row index: `column` - the column index **Returns:**: the cell editor

---


### isReadOnly

```
public boolean isReadOnly()
```

:   returns whether the model is read-only

    :   **Returns:**: true if model is read-only

---


### setReadOnly

```
public void setReadOnly(boolean value)
```

:   sets whether the model is read-only

    :   **Parameters:**: `value` - if true the model is set to read-only

---


### getPlainColumnName

```
public java.lang.String getPlainColumnName(int columnIndex)
```

:   returns the basically the attribute name of the column and not the
    HTML column name via getColumnName(int)

    :   **Parameters:**: `columnIndex` - the column index **Returns:**: the plain name

---


### getStringSelection

```
public java.awt.datatransfer.StringSelection getStringSelection()
```

:   returns the selected content in a StringSelection that can be copied to
    the clipboard and used in Excel, if nothing is selected the whole table
    is copied to the clipboard

    :   **Returns:**: the current selection

---


### setSearchString

```
public void setSearchString(java.lang.String searchString)
```

:   sets the search string to look for in the table, NULL or "" disables
    the search

    :   **Parameters:**: `searchString` - the search string to use

---


### getSearchString

```
public java.lang.String getSearchString()
```

:   returns the search string, can be NULL if no search string is set

    :   **Returns:**: the current search string

---


### setSelectedColumn

```
public void setSelectedColumn(int index)
```

:   sets the selected column

    :   **Parameters:**: `index` - the column to select

---


### tableChanged

```
public void tableChanged(javax.swing.event.TableModelEvent e)
```

:   This fine grain notification tells listeners the exact range of cells,
    rows, or columns that changed.

    :   **Specified by:**: `tableChanged` in interface `javax.swing.event.TableModelListener` **Overrides:**: `tableChanged` in class `javax.swing.JTable`
    :   **Parameters:**: `e` - the table event

---


### addChangeListener

```
public void addChangeListener(javax.swing.event.ChangeListener l)
```

:   Adds a ChangeListener to the panel

    :   **Parameters:**: `l` - the listener to add

---


### removeChangeListener

```
public void removeChangeListener(javax.swing.event.ChangeListener l)
```

:   Removes a ChangeListener from the panel

    :   **Parameters:**: `l` - the listener to remove


---


|  |  |  |
| --- | --- | --- |
| **Overview** \| **Package** \| **Class** \| **Tree** \| **Deprecated** \| **Index** \| **Help** \| **Weka's home** | |  |
| **PREV CLASS**   **NEXT CLASS** | **FRAMES**    **NO FRAMES**     **All Classes** |  |
| SUMMARY: NESTED \| FIELD \| CONSTR \| METHOD | DETAIL: FIELD \| CONSTR \| METHOD |  |


---
